# Do Sleeping Disorders Impair Sexual Function in Married Iranian Women of Reproductive Age? Results from a Cross-Sectional Study

**DOI:** 10.1155/2018/1045738

**Published:** 2018-04-23

**Authors:** Poorandokht Afsahri, Zahra Salehnejad, Khadijeh Hekmat, Parvin Abedi, Ahmad Fakhri, Mohammadhossein Haghighizadeh

**Affiliations:** ^1^Reproductive Health Promotion Research Center, Midwifery Department, Ahvaz Jundishapur University of Medical Sciences, Ahvaz, Iran; ^2^Menopause Andropause Research Center, Midwifery Department, Ahvaz Jundishapur University of Medical Sciences, Ahvaz, Iran; ^3^Psychiatry Department, Reproductive Health Promotion Research Center, Ahvaz Jundishapur University of Medical Sciences, Ahvaz, Iran; ^4^Biostatistics Department, Health Faculty, Ahvaz Jundishapur University of Medical Sciences, Ahvaz, Iran

## Abstract

**Purpose:**

This study aimed to evaluate the relationship between sleep quality and sexual function among Iranian women.

**Methods:**

This study was conducted on 277 married women of reproductive age. The inclusion criteria were as follows: married women aged 18–45 years, with at least basic literacy, and women married monogamously for at least one year. The following tools were used for gathering data: a demographic questionnaire; Pittsburgh Sleep Quality Index (PSQI); Insomnia Severity Index (ISI); Epworth Sleepiness Scale (ESS); and Female Sexual Function Index (FSFI). Pearson correlation coefficients, independent *t*-tests, chi-square tests, and linear regression analyses were used to analyze the data.

**Results:**

There was a significant inverse relation between poor sleep quality (*r* = −0.13, *P* = 0.02), daytime sleepiness (*r* = −0.39, *P* < 0.001), insomnia (*r* = −0.35, *P* < 0.001), and sexual function. Sexual desire was significantly related to sleep quality and insomnia (*P* < 0.001). Sexual arousal (*r* = −0.18, *r* = −0.29, *P* < 0.001), lubrication (*r* = −0.21, *r* = −0.3, −0.12, *P* < 0.001), orgasms (*r* = 0.17, *r* = −0.15, *P* < 0.001), and sexual satisfaction (*r* = −0.02, −*r* = 0.3, *r* = −0.15, *P* < 0.001) were significantly related to all types of sleep disorders (poor sleep quality, insomnia, and sleepiness). Pain during intercourse was significantly associated with poor sleep quality and insomnia. With each unit decrease in sleep quality, sexual function decreased by 0.49 (*P* < 0.001), and with each unit increase in the delay of sleep onset, sexual function decreased by 1.58 (*P* = 0.04).

**Conclusion:**

Results of this study showed that there was a significant relationship between sleep quality and sexual function in Iranian women of reproductive age. The quality of sleep among reproductive-aged women merits the attention of health care providers and policy makers.

## 1. Introduction

Female sexual function consists of the domains of desire, libido, arousal, pain/discomfort, and orgasms [1, 2]. Impairment in any of these domains can prevent the sexual pleasure experienced by couples [3]. According to the International Classification of Disease (ICD-11), sexual activity is a combination of psychological, interpersonal, social, cultural, physiological, and gender characteristics, and impairment in any of these factors may cause sexual dysfunction [4]. According to a large systematic review, the prevalence of female sexual orgasmic disorders globally ranged from 7 to 10% [5]. The prevalence of sexual disorders and sexual arousal disorders among women in Iran is 35% and 33.8%, respectively [6]. Additionally, the prevalence of anorgasmia in women of reproductive age in Iran is reported to be 26.3% [7]. The high prevalence of sexual disorders in Iranian women may be due to the lack of knowledge about sexual issues and misconceptions regarding sexual intimacy [6].

The quality of life of women with sexual dysfunction is lower than that of women without sexual disorders [8]. A study of 784 married women in Iran reported that the prevalence of sexual disorders was 27.3% and that there was a significant relationship between sexual dysfunction and women's reported satisfaction with their routine life [9].

Sexual function is related to many physical and psychological factors, and one such factor is sleep quality. In a study of 54 women of reproductive age with fibromyalgia, Amasyali et al. (2016) determined that the sexual function score was significantly higher in patients with better sleep quality [10]. Another study by Kalmbach et al. (2015) showed that longer sleep duration in women was significantly correlated with higher sexual desire and also could improve engaging in the sexual activity, but it did not have any relationship with the genital arousal [11]. A study including 93,668 pre- and postmenopausal women showed that sleep duration less than 6 hours could significantly reduce sexual activity [12]. Also, there is evidence that shows a relationship between total scores of mental health and sleep quality [13]. Dağ and Kutlu (2017) showed that poor sleep quality significantly contributed to mild and moderate-severe depression among adolescents [14].

Despite the fact that the etiology of sleep or sexual dysfunction may be organic or psychogenic, sleep and sexual activity are both biologically endogenously regulated processes [15]. Studies have shown that women with poor sleep quality and obstructive sleep apnea have lower levels of progesterone, estradiol, and 17-OH-progesterone compared to healthy women [16].

There are some studies that have addressed the relationship between sleep quality and sexual function in men or individuals with other medical disorders [17, 18]. However, there is a paucity of information regarding the relationship between sleep quality and sexual function in healthy reproductive-aged women. Therefore, the aim of this study was to evaluate the relationship between sleep quality and sexual function among reproductive-aged Iranian women.

## 2. Materials and Methods

### 2.1. Study Design

This was a cross-sectional study that was conducted on 277 married women of reproductive age from April to December 2015. The study design was approved by the Ethics Committee of Ahvaz Jundishapur University of Medical Sciences (Ref. number 1394-672 IR.AJUMS.REC). All eligible women provided written informed consent before enrollment.

#### 2.1.1. Study Participants

The inclusion criteria were as follows: married women aged 18–45 years, with at least basic literacy, and women married monogamously for at least one year. We excluded women with medical disorders such as diabetes, hypertension, cancer, multiple sclerosis, thyroid, and psychological disorders, or those who experienced any general critical events in the last 6 months.

Our sample size (*n* = 277) was calculated based on the results of a pilot study conducted on 40 women who met the inclusion criteria and who were admitted to Shahid Mustafa Khomeini Hospital in Behbahan, Iran. Eligible participants were recruited from women who attended the health clinic in the Mustafa Khomeini Hospital in Behbahan for other reasons such as vaginitis, Pap smear screening, and growth monitoring of their children. Women from all parts of Behbahan attended to this clinic. Recruitment of participants was conducted for 6 months. Once consented, eligible women were asked to self-administer all questionnaires, and one of the researchers (ZS) was available there in order to clarify any ambiguity regarding the study questions.

### 2.2. Measures

The following tools were used for data collection: a demographic questionnaire; Pittsburgh Sleep Quality Index (PSQI); Insomnia Severity Index (ISI); Epworth Sleepiness Scale (ESS); Female Sexual Function Index (FSFI).

PSQI contains 19 self-report questions and five questions specifically for the spouse of the woman completing the questionnaire. In this study, we used only the self-report questions. Of these questions, 15 were multiple choices and measured the frequency of subjective sleep problems and sleep quality, while the remaining four were about bed time, time of arousal, the latency period of sleep, and subjective quality of sleep. This questionnaire has seven domains including subjective sleep quality, sleep latency, sleep duration, sleep disturbances, use of sleeping medication, habitual sleep efficiency, and daytime dysfunction. Each domain has a score from zero (without problems) to three (severe problems). The total score of this questionnaire varies from 0 to 21; a score ≥ 5 indicates poor sleep quality. The validity and reliability of this scale were demonstrated by Backhaus et al. [19], and in Iran, Farrahi Moghaddam et al. confirmed its validity and reliability [20].

The ISI questionnaire contains seven questions that measured the participant's perception of the severity of insomnia during the past two weeks. Responses were structured using Likert scales; zero indicated “never” and four indicated “always.” Overall scores of 0–7, 8–14, 15–21, and 22–28 revealed normal sleep patterns, sleep disorder below the threshold, moderate clinical insomnia, and severe clinical insomnia, respectively. The validity and reliability of this questionnaire were demonstrated by Morin et al., in 2011 [21], while the validity and reliability of the Persian version of this questionnaire were demonstrated by Yazdi et al., in Iran [22].

The ESS consists of eight questions that measure the daily sleepiness of participants. Each question was assigned a score between zero and three. The overall score ranged from zero (lack of sleepiness) to 24 (sleepiness in all eight situations). The scores 0–8, 9–12, 13–16, and >16 indicated a normal situation, mild sleepiness, moderate sleepiness, and severe sleepiness, respectively. The validity and reliability of this questionnaire were assessed and confirmed by Johns [23], while the validity and reliability of the Persian version of this questionnaire were assessed by Sadeghniiat Haghighi et al. in Iran [24].

The FSFI contains 19 questions that measure the sexual female function in six separate domains including sexual desire, sexual arousal, lubrication, orgasm, sexual satisfaction, and intercourse pain. Two questions were used for measuring sexual desire, four questions for sexual arousal, four questions for lubrication, and three questions for orgasm, sexual satisfaction, and pain each. The total score for each domain was multiplied by the following factors: sexual desire score by 0.6; sexual arousal and lubrication by 0.3; orgasm, sexual satisfaction, and pain by 0.4. A total score for sexual function ≥26 was considered normal. The validity and reliability of this questionnaire were assessed and confirmed by Rosen et al. [2], while the validity and reliability of the Persian version of this questionnaire were demonstrated by Fakhri et al. [25] in Iran.

### 2.3. Statistical Analyses

We used SPSS version 22 for analysis of our study data. Pearson correlation coefficients were used to measure the relationship between sleep disorders and sexual function. Independent *t*-tests were used for comparisons of continuous data, while chi-square tests were used for comparisons of categorical data. We used stepwise multiple linear regressions analyses to evaluate the relationship between demographic variables and those related to sleep and sexual function. *P* values < 0.05 were considered statistically significant.

## 3. Results

We screened 350 women of whom 277 were eligible and recruited for this study. The sociodemographic characteristics of the participants are presented in Table 1, stratified by sleep quality (good versus poor) based on PSQI score.

The mean ages of women with poor and good sleep quality were 31.37 ± 6.84 and 30.13 ± 6.41 (*P* > 0.05), respectively. Women with poor sleep quality had a significantly longer duration of marriage, a greater age difference with their husband, and a greater number of children. Additionally, there were significant differences between the two groups in terms of education, education level of the spouse, and economic status (*P* < 0.05) (Table 1).

Table 2 shows the differences between the various domains of sexual activity in women with poor versus good sleep quality. All of the domains of sexual activity were significantly lower in women with poor sleep quality compared to those with good sleep quality (*P* < 0.05). The total mean score of FSFI was 22.6 ± 9.8 and 25.7 ± 7.8 in the women with poor sleep quality versus those with good sleep quality, respectively (*P* = 0.004, Table 2).

Table 3 shows the relationship between sleep disturbances and sexual function in married women. There was inverse relationship between sexual function and subjective sleep quality (*r* = −0.13, *P* = 0.02), sleep latency (*r* = −0.35, *P* < 0.001), sleep duration (*r* = −0.18, *P* < 0.001), sleep disturbances (*r* = −0.22, *P* < 0.001), daytime function (*r* = −0.39, *P* < 0.001), global PSQI score (*r* = −0.40, *P* < 0.001), insomnia (*r* = −0.35, *P* < 0.001), and sleepiness (*r* = −0.15, *P* = 0.01) (Table 3).

Table 4 depicts the relationship between types of sleep disorders and sexual function domains using the PSQI, ISI, and ESS questionnaires. Sexual desire was significantly related to sleep quality and insomnia (*P* < 0.001). Additionally, sexual arousal, lubrication, orgasms, and sexual satisfaction were significantly related to all types of sleep disorders (sleep quality disturbances, insomnia, and sleepiness) (*P* < 0.05). Pain during intercourse was significantly related to only sleep quality disturbances and insomnia (Table 4).

The results of the present study indicated that overall 151 (55.9%) of the participants had poor sleep quality and 126 (46.6%) had good sleep quality (based on PSQI questionnaire). Also, results of ISI questionnaire revealed that 127 (45.8%) of the women had normal sleep pattern, 96 (34.7%) had sleep disorder below the threshold, 45 (16.2%) had moderate clinical insomnia, and 8 (2.9%) had severe clinical insomnia. Analysis of ESS questionnaire showed that 184 (66.4%) of women did not have any sleepiness, 53 (19.1%) had mild sleepiness, 35 (12.6%) had moderate sleepiness, and 5 (1.8%) had severe sleepiness. Based on FSFI, 139 (50.2%) of the participants had sexual dysfunction and 138 (49.8%) had normal sexual function (data are not presented in tables). None of participants used medication for sleep.

Table 5 indicates the relationship between poor sleep quality, age, and delay on sleep onset and sexual function using stepwise multiple linear regressions analyses. With each unit decrease in sleep quality, sexual function decreased by 0.49 (*P* < 0.001). With each unit increase in the age of participants, sexual function increased by 0.18 (*P* = 0.01). Finally with each unit increase in the delay to sleep onset, sexual function decreased by 1.58 (*P* = 0.04). We found that if the effects of age, sleep quality disorders, and delay in sleep onset were eliminated, the sexual function score would increase by 33.92.

## 4. Discussion

This study aimed to evaluate the relationship between sleep quality and sexual function among reproductive-aged women. We found that there was a significant inverse relationship between sleep quality and sexual function. Also, there was a significant relationship between all types of sleep quality (sleep quality, insomnia, and sleepiness) and sexual arousal, lubrication, orgasms, and sexual satisfaction. Pain during intercourse and sexual desire were significantly associated with only sleep quality and insomnia.

Previous studies have shown that sexual dysfunction is quite prevalent among Iranian reproductive-aged women. Our results also indicated that 50.2% of the women participating in our study had sexual dysfunction according to total score of FSFI. A study on 784 Iranian women showed that approximately 27% had sexual dysfunction, with a lack of sexual desire being the most prevalent type (35.6%) [9]. Another study of 1200 Iranian women showed that 26.3% had anorgasmia [7]. A study in Hong Kong on 1,518 women aged 21–49 showed that at least 25.6% of women reported one form of sexual dysfunction [26]. Also, in a systematic review including 95 studies conducted in different countries, McCool et al. found that the prevalence of sexual dysfunction in 40.9% of premenopausal women, with a higher prevalence in African women [27].

 Kuka conducted a study on 260 female students with the aim of assessing the associations between sleep quality, sexual function, stress, medical conditions, and the use of prescription medications [15]. Results showed that there was a positive nonsignificant relationship between sleep quality and sexual function, but not between stress and sleep quality. The discrepancies between our results and those of Kuka may have resulted from the fact that we did not measure the stress levels of the enrolled participants.

Amasyali et al. conducted a study on 54 women of reproductive age with fibromyalgia to assess their sexual activity. They found that the total sexual function score was significantly lower in women with poor sleep quality compared with those with good sleep quality (median: 29.2 versus 21.4, *P* < 0.001) [10]. These results are in line with our findings, which demonstrated that women with poor sleep quality had a lower total sexual function score compared with women without sleep disorders. Similar to our study, another study which included 210 men and women and used the PSQI showed that women with poor sleep quality were more susceptible to psychosocial distress, higher blood sugar, and expression of inflammatory biomarkers [28].

One of the underlying hypotheses for the effect of sleep disturbances on sexual function is the activation of the hypothalamic-pituitary-adrenal axis (HPA). Also, circadian dysrhythmia itself or in combination with HPA may interfere with sexual activity and reproductive health possibly even leading to infertility [29]. Circadian rhythms are endogenous rhythms that occur over a 24-hour period that are related to the physical environment and affected by photic and nonphotic stimuli. Disorders in circadian rhythms may result in sleep onset or sleep maintenance and excessive sleepiness [30].

One study showed that there is a relationship between chronic insomnia and the activity of the stress system. In this study the levels of urinary free cortisol, catecholamines, and Growth Hormones were measured in insomniacs, and the investigation found that the level of urinary free cortisol was positively correlated with the total time awake [31]. Studies have also shown that higher levels of cortisol, fatigue, and stress are underlying factors driving sexual dysfunction [32, 33]. Mental disorders such as anxiety may reduce lubrication and clitoral vascular congestion and result in reduction of sexual arousal [34]. Also women with depression experience more sexual dysfunction [35]. Reduced sleep quality may be a cause of sleepiness during the day [36], which may result in accidents, decreased attention, alertness, and concentration, and reduced sexual drive [37].

Our results showed that, with each unit decrease in sleep quality, sexual function decreased by 0.49, while with each unit increase in the age of participants, sexual function increased by 0.18. A review study showed that, with increasing age, sexual function will decrease; however, this decline only begins in participants in their twenties to late thirty [38]. In the present study, since most participants aged between 30 and 31 years, we did not investigate the association between aging and reduced sexual function. Kling et al. [12] conducted a longitudinal study on 171 women to assess the effect of nighttime sleep duration, sleep quality, and sleep onset latency on daily female sexual activity. Results showed that a longer duration of sleep could increase next-day's sexual desire and activity and improve genital arousal (*P* < 0.05). These results are in line with our findings.

## 5. Strength and Limitations

To the best of our knowledge, this is the first study to assess the relationship between sexual function and sleep quality in Iran. We used three standard questionnaires for measuring sleep quality in the enrolled women. Despite the strengths of this study, it was subject to several limitations. First, we did not recruit women randomly, which limit the generalizability of our findings. Furthermore, we did not ask the women's spouses about their wives' quality of sleep nor did we measure the sexual function of the male partner. Finally, we did not assess the psychological state of the participants objectively such as using Beck or Stress questionnaires. Instead, we asked the women about this, and if they mentioned any psychological disorders or stress, we excluded them.

## 6. Conclusion

Results of this study showed that there was a significant relationship between sleep quality and sexual function in reproductive age Iranian women. Therefore, the quality of sleep among reproductive-aged women merits the attention of health care providers and policy makers.

## Figures and Tables

**Table 1:** Sociodemographic characteristics of women with poor sleep quality and good sleep quality.

Variable	Poor sleep quality (PSQI score ≥ 5) *n* = 151	Good sleep quality (PSQI < 5) *n* = 126	Test value	*P* value
	*Mean ± SD*			
Age (y)	31.37 ± 6.84	30.13 ± 6.41	*t* = −1.42	0.15
Age at marriage (y)	22.15 ± 4.53	24.76 ± 4.43	*t* = 3.09	<0.001
Duration of marriage (y)	9.19 ± 9.25	5.49 ± 6.56	*t* = −3.15	<0.001
Age difference with spouse (y)	5.05 ± 3.52	4.86 ± 3.13	*t* = −0.46	0.64
Number of children	1.85 ± 1.30	1.23 ± 1.11	*t* = −3.23	<0.001
	*N (%)*			
*Education *				
High school	19 (12.59)	10 (7.94)	*χ* ^2^ = 37.07	<0.001
Diploma	57 (37.75)	29 (23.02)		
University education	75 (49.67)	87 (69.05)		
*Education of spouse *				
High school	23 (15)	8 (6.35)	*χ* ^2^ = 5.64	0.01
Diploma	51 (34)	31 (24.61)		
University education	77 (51)	87 (69.05)		
*Mode of delivery *				
Normal vaginal delivery	65 (43.05)	42 (33.34)	*χ* ^2^ = 0.12	0.79
Cesarean section	51 (33.78)	40 (31.75)		
Both vaginal and cesarean section	2 (1.33)	4 (3.18)		
Nulliparous	33 (21.86)	40 (31.75)		
*Economic situation *				
Weak	26 (17.22)	7 (5.56)	*χ* ^2^ = 5.55	<0.001
Moderate	67 (44.37)	51 (40.48)		
Good	55 (36.43)	63 (50)		
Well off	3 (1.99)	5 (3.97)		

Data analyzed using independent *t*-test or chi-square. Women classified in two groups of poor sleep quality and good sleep quality according to PSQI questionnaire.

**Table 2:** Different domains of sexual activity in women with poor and good sleep quality.

Variables	Poor sleep quality (PSQI ≥ 5) *n* = 151	Good sleep quality (PSQI < 5) *n* = 126	*P* value
	Mean ± SD		
Desire	3.39 ± 1.06	3.98 ± 1.08	<0.001
Arousal	3.34 ± 1.54	3.97 ± 1.56	0.001
Lubrication	3.64 ± 1.52	4.22 ± 1.44	0.001
Orgasm	3.84 ± 1.71	4.43 ± 1.49	0.003
Satisfaction	3.95 ± 1.84	4.71 ± 1.62	<0.001
Dyspareunia	4.03 ± 1.71	4.61 ± 1.59	0.005
FSFI	22.6 ± 9.8	25.7 ± 7.8	0.004

Women classified in two groups of poor sleep quality and good sleep quality according to PSQI questionnaire. Data analyzed using independent *t*-test.

**Table 3:** The relation of sleep quality with sexual function in women.

Variables	Spearman coefficient	*P* value
Subjective sleep quality^*∗*^	−0.13	0.02
Sleep latency^*∗*^	−0.35	<0.001
Sleep duration^*∗*^	−0.18	<0.001
Sleep disturbances^*∗*^	−0.22	<0.001
Daytime function^*∗*^	−0.39	<0.001
Global PSQI score^*∗∗*^	−0.40	<0.001
Insomnia^∞^	−0.35	<0.001
Sleepiness^Ω^	−0.15	0.01

^*∗*^Items related to PSQI questionnaire. ^*∗∗*^Total score of PSQI questionnaire. ^∞^Total score of ISI questionnaire. ^Ω^Total score of ESS questionnaire. Data analyzed using Spearman correlation test.

**Table 4:** The relation of sleep quality and sleep disturbances with sexual function domains.

Variables	Sleep quality (PSQI)	Insomnia severity (ISI)	Sleepiness (ESS)
Sexual desire	−0.17^*∗*^	−0.29^*∗*^	−1
Sexual arousal	−0.18^*∗*^	−0.35^*∗*^	−0.13^*∗∗*^
Lubrication	−0.21^*∗*^	−0.3^*∗*^	−0.12^*∗∗*^
Orgasm	0.17^*∗*^	−0.3	−0.15^*∗∗*^
Sexual satisfaction	−.021^*∗*^	−0.3^*∗*^	−0.15^*∗∗*^
Pain	−0.19^*∗*^	−0.30^*∗∗*^	−0.07

^*∗*^ *P* < 0.001;  ^*∗∗*^*P* < 0.05. Data analyzed using Spearman correlation.

**Table 5:** The multiple linear regression analysis for assess relation of sexual function with some variables.

	Variables	*β*	SE	*P* value
(1)	Intercept	28.21	0.94	<0.001
	Poor sleep quality	−7.48	0.14	<0.001

(2)	Intercept	33.44	2.55	<0.001
	Poor sleep quality	−7.22	0.14	<0.001
	Age	−0.17	0.07	<0.001

(3)	Intercept	33.92	2.55	<0.001
	Poor sleep quality	−0.49	0.18	<0.001
	Age	0.18	0.07	0.01
	Sleep latency	−1.58	0.07	0.04

Data analyzed using linear regression.
